# Supplementary figures and images for: Meteorological drought under historical and future climate scenarios in North Gojjam sub-basin, Abay River basin of Ethiopia
Source: PLoS One. 2025 Jul 17;20(7):e0328105. doi: 10.1371/journal.pone.0328105 (PMC12270152; doi:10.1371/journal.pone.0328105)

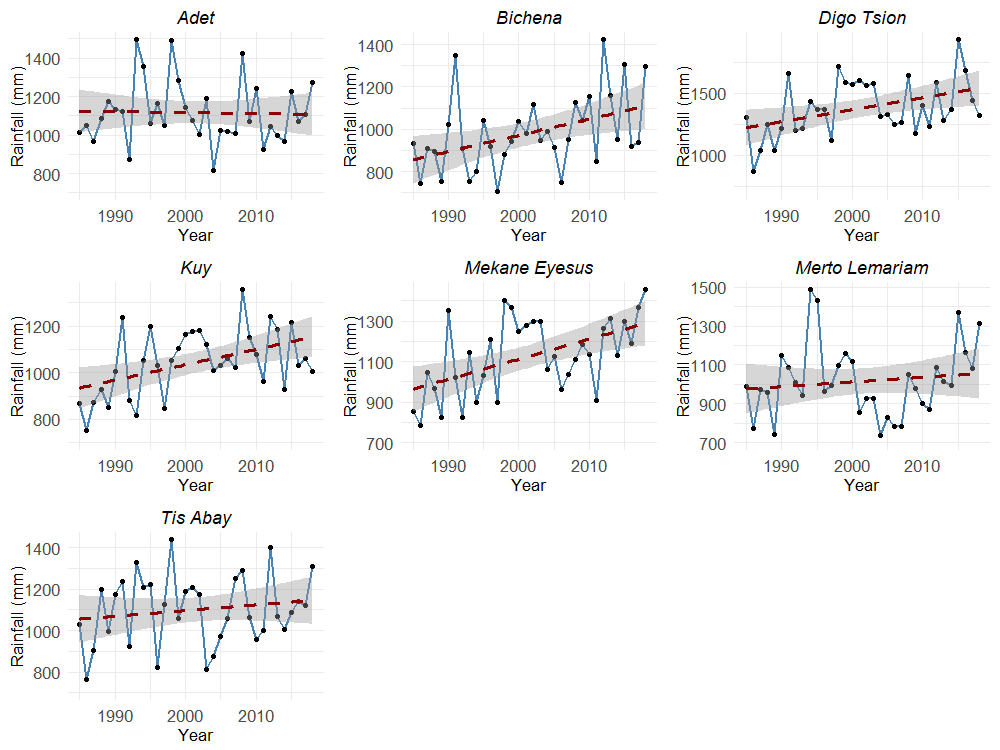


**S1 Fig.** Annual rainfall trends for seven locations between 1985 and 2018.

Supplement: S1 Fig — (DOCX) [file pone.0328105.s001.docx]

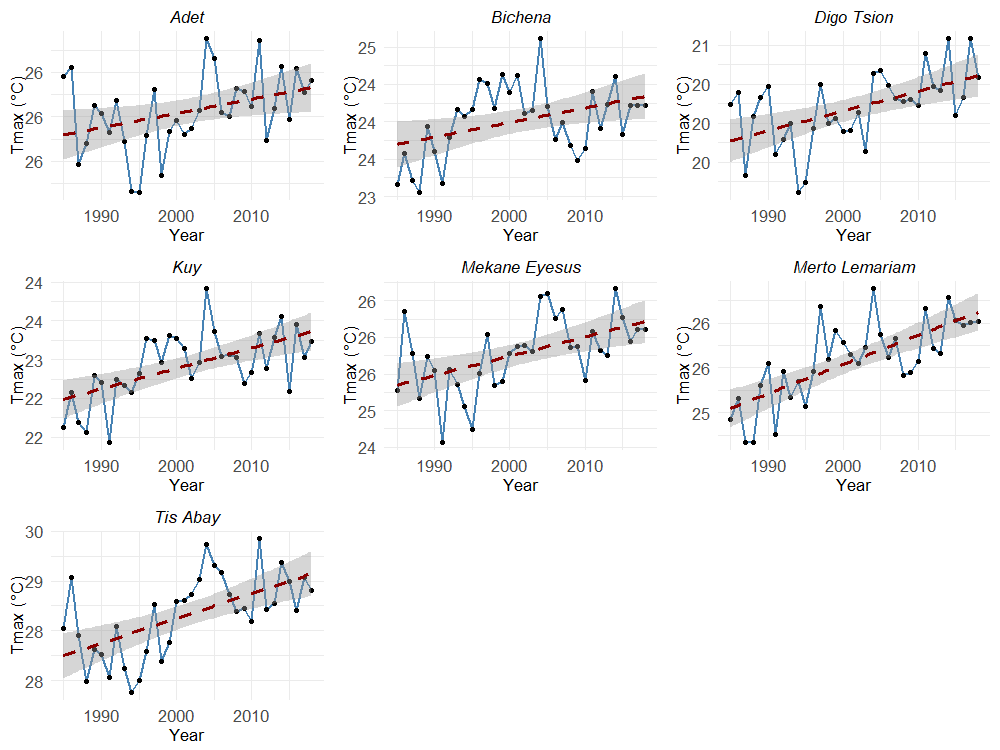


**S2 Fig.** Mean annual maximum Temperature trends for seven locations between 1985 and 2018

Supplement: S2 Fig — (DOCX) [file pone.0328105.s002.docx]

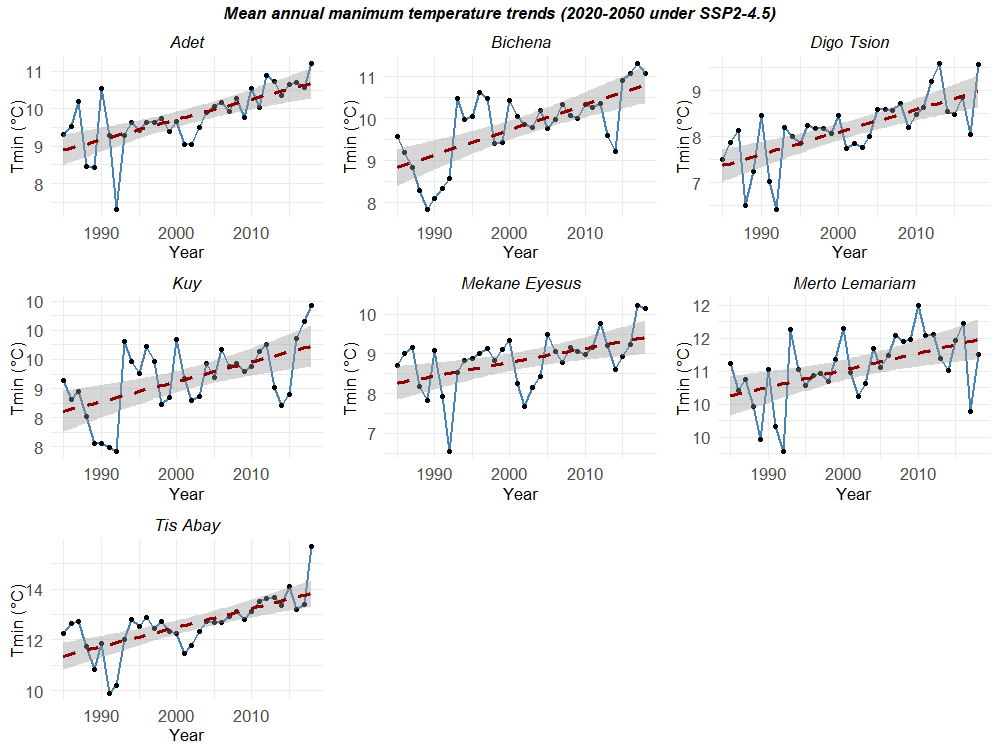


**S3 Fig.** Mean annual minimum temperature trends for seven locations between 1985 and 2018

Supplement: S3 Fig — (DOCX) [file pone.0328105.s003.docx]

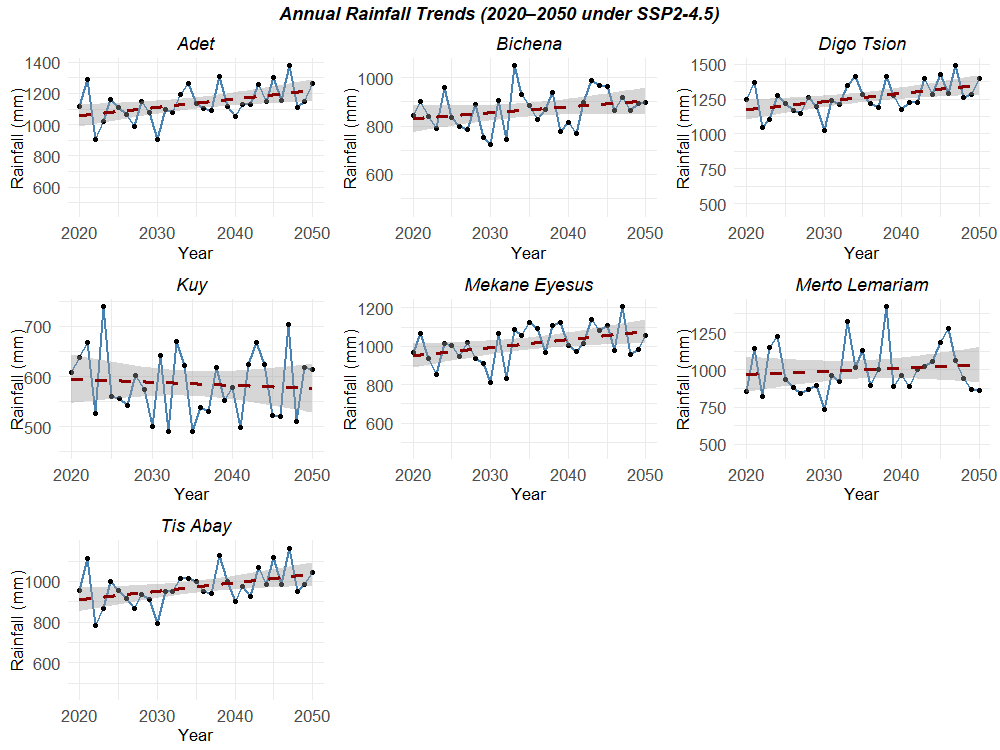


**S4 Fig.** Annual rainfall trends for seven locations from 2020 to 2050 under the SSP2-4.5 climate scenario

Supplement: S4 Fig — (DOCX) [file pone.0328105.s004.docx]

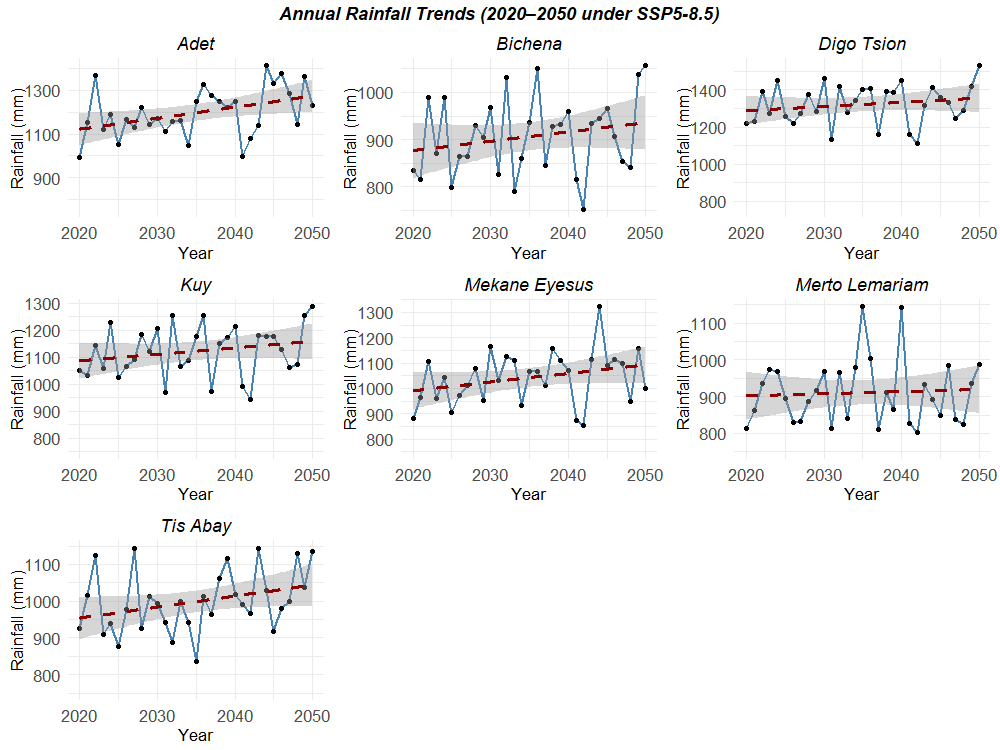


**S7 Fig.** Annual rainfall trends for seven locations from 2020 to 2050 under the SSP5-8.5 climate scenario

Supplement: S7 Fig — (DOCX) [file pone.0328105.s007.docx]

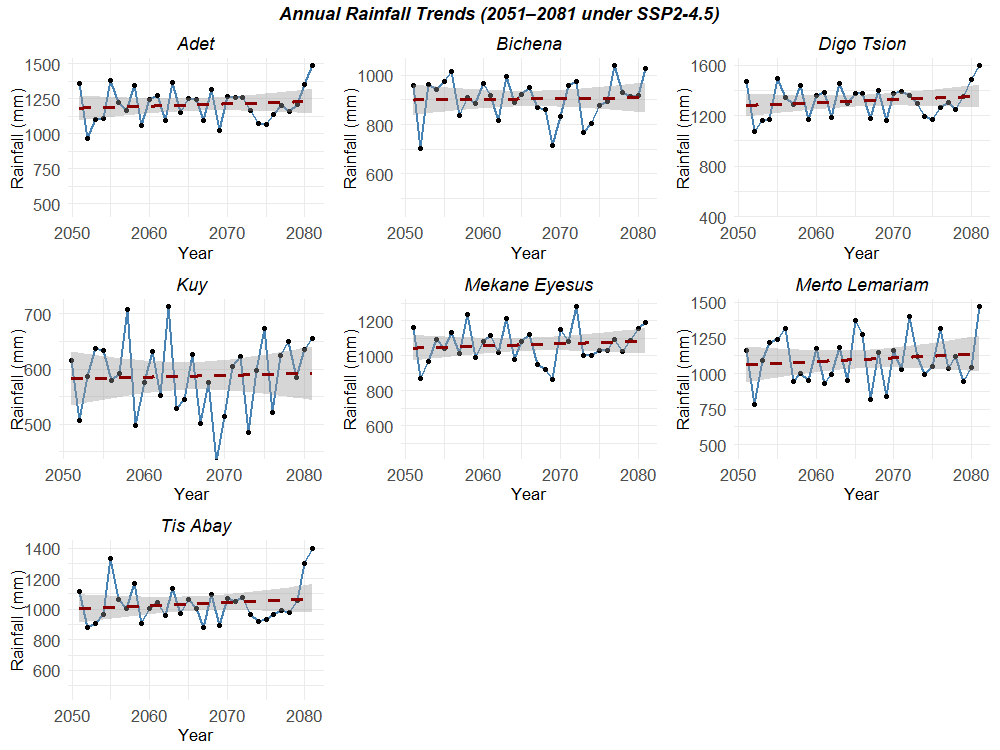
**S10 Fig.** Annual rainfall trends for seven locations from 2051 to 2081 under the SSP2-4.5 climate scenario

Supplement: S10 Fig — (DOCX) [file pone.0328105.s010.docx]

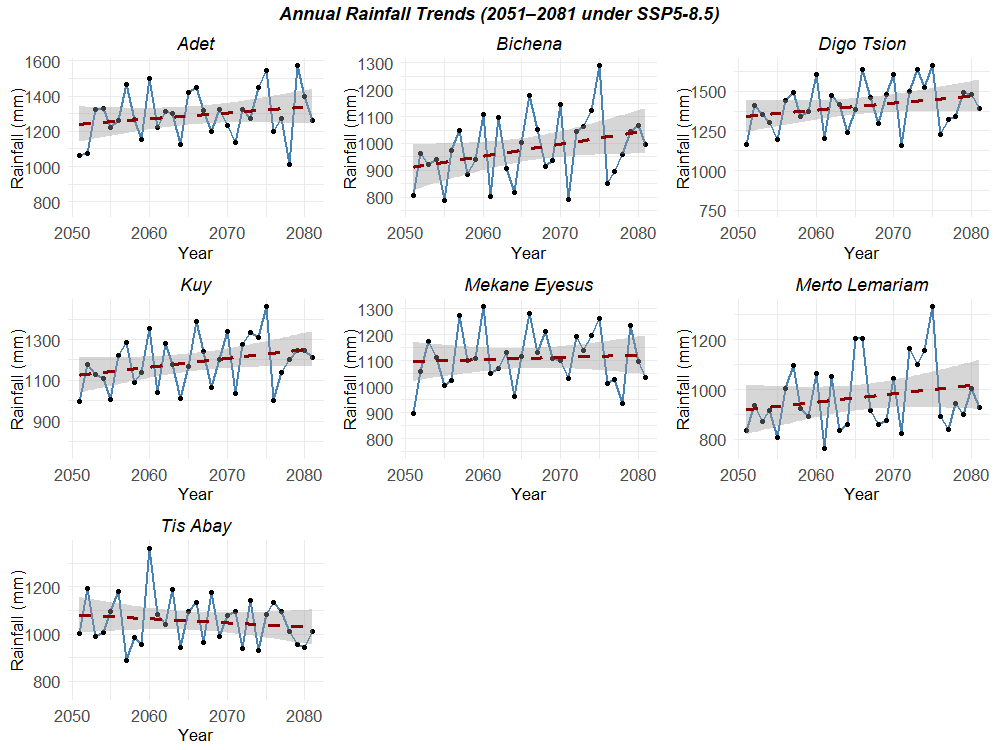


**S13 Fig.** Annual rainfall trends for seven locations from 2051 to 2081 under the SSP5-8.5 climate scenario

Supplement: S13 Fig — (DOCX) [file pone.0328105.s013.docx]
