# Supplementary material for: Meteorological drought under historical and future climate scenarios in North Gojjam sub-basin, Abay River basin of Ethiopia
Source: PLoS One. 2025 Jul 17;20(7):e0328105. doi: 10.1371/journal.pone.0328105 (PMC12270152; doi:10.1371/journal.pone.0328105)
Supplement: S8 Fig — (DOCX) [file pone.0328105.s008.docx]

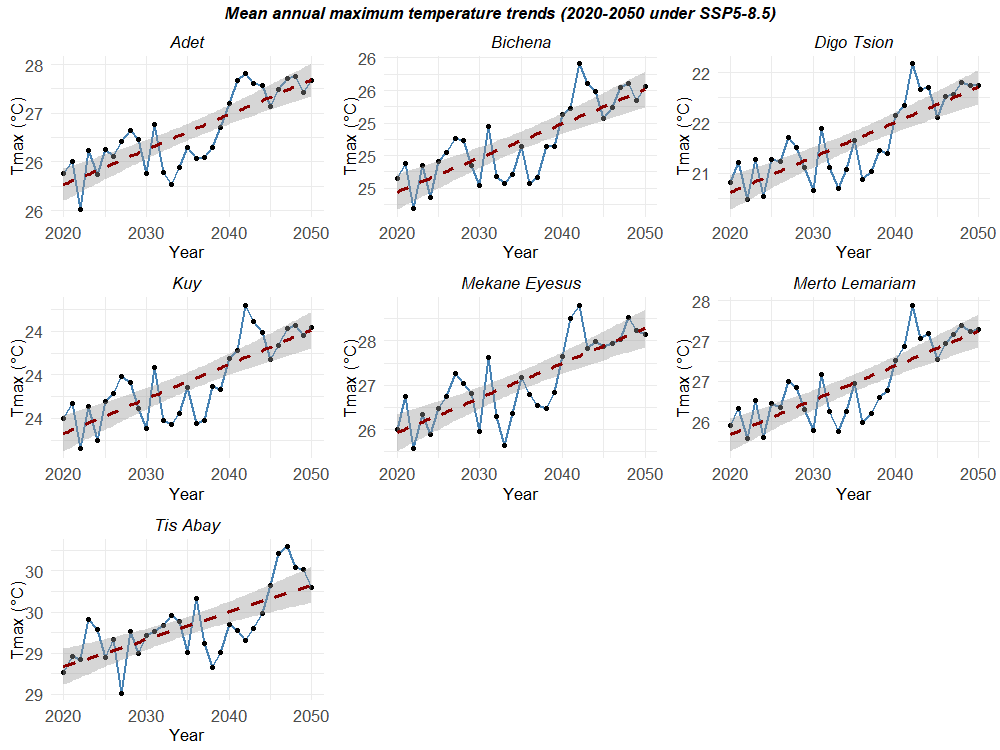


**S8 Fig.** Mean annual maximum temperature trends for seven locations from 2020 to 2050 under the SSP5-8.5 climate scenario
