# Supplementary material for: Meteorological drought under historical and future climate scenarios in North Gojjam sub-basin, Abay River basin of Ethiopia
Source: PLoS One. 2025 Jul 17;20(7):e0328105. doi: 10.1371/journal.pone.0328105 (PMC12270152; doi:10.1371/journal.pone.0328105)
Supplement: Attachment — Submitted filename: Response to Reviewers.docx [file pone.0328105.s017.docx]

**Response to editor and reviewers on “Meteorological drought under historical and future climate scenarios in North Gojjam sub-basin, Abay River basin of Ethiopia”**

**Manuscript Number**: PONE-D-24-44303

**Dear Editor**

Thank you for giving me the opportunity to submit a revised draft of our manuscript titled **Meteorological drought under historical and future climate scenarios in North Gojjam sub-basin, Abay River basin of Ethiopia** to *PLOS ONE.*  We appreciate the time and effort that you and the reviewers have dedicated to providing your valuable feedback on our manuscript. We are grateful to the reviewers for their insightful comments on our paper. We have been able to incorporate changes to reflect most of the suggestions provided by the reviewers. We have highlighted the changes within the manuscript.

**Academic editor comments:** The manuscript needs to be improved significantly by implementing reviewers' comments. Please provide a detailed point-by-point response file in addition to the revised manuscript. Moreover, I also agree with one of the reviewer comments about using CMIP6. Indeed, adopting a recent version of climate projections can provide a better perspective. Thus, I recommend using CMIP6 instead of CMIP5 unless you have a rational reason for not using it.

**Response:** Dear Academic Editor,

Thank you for your constructive comments and suggestions, as well as for considering this manuscript for publication in the high-impact journal PLOS ONE. Based on your and the reviewers' feedback, we have made the following revisions:

- We have replaced CMIP5 with CMIP6.
- The number of stations used in the analysis has been updated to seven due to similarities in the minimum and maximum temperature and precipitation data extracted from the stations using GCMs. As a result of these changes, the analysis section has also been revised to reflect the use of CMIP6.

**Comment 2:** Please include the following items when submitting your revised manuscript:

A rebuttal letter that responds to each point raised by the academic editor and reviewer(s). You should upload this letter as a separate file labeled 'Response to Reviewers'.

A marked-up copy of your manuscript that highlights changes made to the original version. You should upload this as a separate file labeled 'Revised Manuscript with Track Changes'.

An unmarked version of your revised paper without tracked changes. You should upload this as a separate file labeled 'Manuscript'.

If you would like to make changes to your financial disclosure, please include your updated statement in your cover letter. Guidelines for resubmitting your figure files are available below the reviewer comments at the end of this letter.

Please reference all numbered equations in text. Currently, numbered equations [1] in the manuscript have not been cited in text.

Response: Dear editor, Thank you for your comments. We have addressed the points raised by the academic editor and reviewers, and our detailed responses are attached as a separate file for your reference. Additionally, we have submitted both a clean version of the manuscript (without tracked changes) and a revised version with tracked changes, as per your request. All equations have now been properly cited within the text.

**Editor and Reviewer comments:**

**Comment 1:** Journal requirements: When submitting your revision, we need you to address these additional requirements. Please ensure that your manuscript meets PLOS ONE's style requirements, including those for file naming.

Response 1: Thank you for your comments. We have reviewed the requirements of PLOS ONE, including those for file names, and have made corrections accordingly to ensure compliance with the journal's guidelines.

Comment 2: When completing the data availability statement of the submission form, you indicated that you will make your data available on acceptance. We strongly recommend all authors decide on a data sharing plan before acceptance, as the process can be lengthy and hold up publication timelines. Please note that, though access restrictions are acceptable now, your entire data will need to be made freely accessible if your manuscript is accepted for publication. This policy applies to all data except where public deposition would breach compliance with the protocol approved by your research ethics board. If you are unable to adhere to our open data policy, please kindly revise your statement to explain your reasoning and we will seek the editor's input on an exemption. Please be assured that, once you have provided your new statement, the assessment of your exemption will not hold up the peer review process.

Response 2: Thank you for your comments. I acknowledge and accept the PLOS ONE data policy and agree to share the data used in this study upon acceptance of the manuscript.

Comment 3. We note that Figure 1 in your submission contain [map/satellite] images which may be copyrighted. All PLOS content is published under the Creative Commons Attribution License (CC BY 4.0), which means that the manuscript, images, and Supporting Information files will be freely available online, and any third party is permitted to access, download, copy, distribute, and use these materials in any way, even commercially, with proper attribution. For these reasons, we cannot publish previously copyrighted maps or satellite images created using proprietary data, such as Google software (Google Maps, Street View, and Earth). For more information, see our copyright guidelines: <http://journals.plos.org/plosone/s/licenses-and-copyright>. We require you to either (1) present written permission from the copyright holder to publish these figures specifically under the CC BY 4.0 license, or (2) remove the figures from your submission:

a. You may seek permission from the original copyright holder of Figure 1 to publish the content specifically under the CC BY 4.0 license.

We recommend that you contact the original copyright holder with the Content Permission Form (<http://journals.plos.org/plosone/s/file?id=7c09/content-permission-form.pdf>) and the following text:

“I request permission for the open-access journal PLOS ONE to publish XXX under the Creative Commons Attribution License (CCAL) CC BY 4.0 (<http://creativecommons.org/licenses/by/4.0/>). Please be aware that this license allows unrestricted use and distribution, even commercially, by third parties. Please reply and provide explicit written permission to publish XXX under a CC BY license and complete the attached form.”

Please upload the completed Content Permission Form or other proof of granted permissions as an ""Other"" file with your submission.

In the figure caption of the copyrighted figure, please include the following text: “Reprinted from [ref] under a CC BY license, with permission from [name of publisher], original copyright [original copyright year].”

b. If you are unable to obtain permission from the original copyright holder to publish these figures under the CC BY 4.0 license or if the copyright holder’s requirements are incompatible with the CC BY 4.0 license, please either i) remove the figure or ii) supply a replacement figure that complies with the CC BY 4.0 license. Please check copyright information on all replacement figures and update the figure caption with source information. If applicable, please specify in the figure caption text when a figure is similar but not identical to the original image and is therefore for illustrative purposes only.

The following resources for replacing copyrighted map figures may be helpful:

USGS National Map Viewer (public domain): <http://viewer.nationalmap.gov/viewer/>

The Gateway to Astronaut Photography of Earth (public domain): <http://eol.jsc.nasa.gov/sseop/clickmap/>

Maps at the CIA (public domain): <https://www.cia.gov/library/publications/the-world-factbook/index.html> and <https://www.cia.gov/library/publications/cia-maps-publications/index.html>

NASA Earth Observatory (public domain): <http://earthobservatory.nasa.gov/>

Landsat: <http://landsat.visibleearth.nasa.gov/>

USGS EROS (Earth Resources Observatory and Science (EROS) Center) (public domain): [http://eros.usgs.gov/#](http://eros.usgs.gov/)

Natural Earth (public domain): <http://www.naturalearthdata.com/>

Response 3: Thank you for your comments. The map presented in Figure 1 is original and has not been published elsewhere. It was created specifically for this study using publicly available and open-source data. I confirm that it fully complies with the requirements of the Creative Commons Attribution License (CC BY 4.0).

**Reviewers' comments:**

Reviewer's Responses to Questions

**Comments to the Author**

1. Is the manuscript technically sound, and do the data support the conclusions?
The manuscript must describe a technically sound piece of scientific research with data that supports the conclusions. Experiments must have been conducted rigorously, with appropriate controls, replication, and sample sizes. The conclusions must be drawn appropriately based on the data presented.

Reviewer #1: Yes

Reviewer #2: Yes

Reviewer #3: No

Response 1: Dear Reviewers, we sincerely thank you for your time, appreciation, and constructive comments, which have been invaluable for improving the manuscript.

2. Has the statistical analysis been performed appropriately and rigorously?

Reviewer #1: Yes

Reviewer #2: Yes

Reviewer #3: No

Response 2: Dear reviewers, we greatly appreciate your time, thoughtful feedback, and constructive comments, which have significantly contributed to enhancing the manuscript.

3. Have the authors made all data underlying the findings in their manuscript fully available?

The [PLOS Data policy](http://track.editorialmanager.com/CL0/http:%2F%2Fwww.plosone.org%2Fstatic%2Fpolicies.action%23sharing/1/010f0193a8579273-4d5c8aa5-643d-4888-93f9-b4395e890b0b-000000/WiO6zEyXW3SmXP2a_6ZH_t7PxSSpZEgdTdXRc-wuJyI=188) requires authors to make all data underlying the findings described in their manuscript fully available without restriction, with rare exception (please refer to the Data Availability Statement in the manuscript PDF file). The data should be provided as part of the manuscript or its supporting information, or deposited to a public repository. For example, in addition to summary statistics, the data points behind means, medians and variance measures should be available. If there are restrictions on publicly sharing data—e.g. participant privacy or use of data from a third party—those must be specified.

Reviewer #1: Yes

Reviewer #2: No

Reviewer #3: Yes

Response 3: Dear Reviewers, thank you for your valuable time and constructive feedback to improve the manuscript.

4. Is the manuscript presented in an intelligible fashion and written in standard English?
PLOS ONE does not copyedit accepted manuscripts, so the language in submitted articles must be clear, correct, and unambiguous. Any typographical or grammatical errors should be corrected at revision, so please note any specific errors here.

Reviewer #1: Yes

Reviewer #2: Yes

Reviewer #3: No

Response 4: Dear Reviewers, thank you for your valuable time and constructive feedback to improve the manuscript.

5. Review Comments to the Author

Please use the space provided to explain your answers to the questions above. You may also include additional comments for the author, including concerns about dual publication, research ethics, or publication ethics. (Please upload your review as an attachment if it exceeds 20,000 characters).

**Comments from Reviewer 1#**

Comment 1: I have the reviewed the manuscript entitled "Meteorological drought under historical and future climate scenarios in North Gojjam sub-basin, Abay River basin of Ethiopia". The whole paper is clear and the different sections are explained in a very detailed and clear way. The manuscript is within the scope of the journal. The methods are sound and the results are consistent with the methods. However the following needs to be addressed: In Figure 1, the map in the top right corner is too small. It needs to be enlarged because the lettering overlaps. I would also make a clearer outline of the basin, the image below where you see the basin has the watershed cut out. It definitely needs to be redone.

Figure 2 needs to be enlarged. Also, the x-axis is truncated in almost all the subplots, and you can't read 2020 in the pictures on the right. Overall, the manuscript is of good scientific quality.

Response 1: Thank you for taking the time to review our manuscript and for your positive feedback. We have revised Figures 1 and 2 in the updated manuscript to enhance their clarity and visibility (see the maps).

Reviewer #2: The article "Meteorological drought under historical and future climate scenarios in North Gojjam sub-basin, Abay River basin of Ethiopia" discovers the future possibility of drought in the Gojjam sub-basin which is important for the region. The article can be improved by considering following comments.

Provide a flow chart summarising the entire methodology adopted.
Include some recent literature from different part of the world such as <https://link.springer.com/article/10.1007/s11269-024-03959-w>
Provide basic equations of trend test. Please provide conclusion in point wise manner.

Response 2: Dear reviewer, Thank you for your valuable comment and suggestion. We have now included a summary of the entire methodology in the revised manuscript based on your suggestion. Please refer to the updated version for details.

Reviewer #3: Introduction:

Comment #1: The introduction acknowledges the inherent uncertainties in climate models but does not specify how these uncertainties will be addressed or quantified. give the results of uncertainties.

Response 1: Thank you for your comment. We have now revised the introduction section in the updated manuscript.

Comment #2: While CMIP5 is a widely used dataset, it has been partially superseded by CMIP6, which includes updated scenarios and improved model simulations. Not using the latest data could limit the study's relevance to current and future research contexts.

Response 2: Dear reviewer, Thank you for your valuable comments. We have now used CMIP6 instead of CMIP5, which has resulted in modifications to the analysis section of the manuscript based on the updated data. The comments have been addressed and incorporated into the revised manuscript. Please find the updated version for your review.

Comment #3: The introduction focuses heavily on Ethiopia but lacks a broader comparison or connection to global drought trends. Adding context about how findings in North Gojjam might contribute to a global understanding of drought dynamics would strengthen the study's appeal.
While the study aims to understand meteorological droughts, it overlooks the implications for agriculture, water resources, or livelihoods. Addressing these aspects would enhance the practical relevance of the research.

Response 3: Dear reviewer, Thank you for your comment. The implications of the study for agricultural water resource management and livelihoods have been incorporated into the updated manuscript.
Methods:
SPEI relies on the accuracy of temperature and precipitation data, which might be prone to uncertainties in data-scarce regions. The use of a log-logistic distribution assumes that the dataset follows this distribution, which might not always hold true. Including these limitations would provide a more balanced perspective. The equations are essential, but their presentation could be simplified. Including explanations for each variable in the text rather than relying on standalone equations would make the methodology more accessible to readers unfamiliar with the HS equation or log-logistic functions. The explanation of how extraterrestrial radiation (Ra) is derived (e.g., through Julian days and latitude) is brief and could confuse readers. A short elaboration or reference to a supplementary figure/table detailing Ra calculation would help. The choice of time scales for SPEI calculation (monthly, annual, multi-scale) is not explicitly provided. Why were these time scales selected, and how do they align with the study’s objectives? Addressing this would strengthen the methodological rationale. While the methodology for calculating SPEI is not well-detailed, it is unclear how this is integrated with CMIP5 data to assess future drought conditions. A brief discussion of how SPEI calculations are adapted for future climate scenarios would bridge the gap between the methods and the study's aims.

Response: Thank you for your constructive comments. We have thoroughly revised the manuscript in line with your suggestions.

The section uses terms like "water surplus," "water deficit," "atmospheric evaporative demand," and "water balance." While these are accurate, their relationship to each other and to SPEI calculation could be clarified for consistency. Some sentences are dense and could benefit from rephrasing for better readability (e.g., lines 211–214). Break down the paragraph into smaller, logically organized sections with subheadings (e.g., "SPI and Its Limitations," "SPEI Calculation," "HS Equation for PET," etc.).

6. PLOS authors have the option to publish the peer review history of their article ([what does this mean?](https://track.editorialmanager.com/CL0/https:%2F%2Fjournals.plos.org%2Fplosone%2Fs%2Feditorial-and-peer-review-process%23loc-peer-review-history/1/010f0193a8579273-4d5c8aa5-643d-4888-93f9-b4395e890b0b-000000/uP3wlo1UOttfGdm9Ime0OYVBG61q4zKAFcVX_yrYKkM=188)). If published, this will include your full peer review and any attached files.
If you choose “no”, your identity will remain anonymous but your review may still be made public.
**Do you want your identity to be public for this peer review?** For information about this choice, including consent withdrawal, please see our [Privacy Policy](https://track.editorialmanager.com/CL0/https:%2F%2Fwww.plos.org%2Fprivacy-policy/1/010f0193a8579273-4d5c8aa5-643d-4888-93f9-b4395e890b0b-000000/Da4Fv4xPj6LSfyUy0lByyiFEGqROhnyDEMzfyI1Xh0o=188).

Reviewer #1: No

Reviewer #2: No

Reviewer #3: No
