# Supplementary material for: Meteorological drought under historical and future climate scenarios in North Gojjam sub-basin, Abay River basin of Ethiopia
Source: PLoS One. 2025 Jul 17;20(7):e0328105. doi: 10.1371/journal.pone.0328105 (PMC12270152; doi:10.1371/journal.pone.0328105)
Supplement: Attachment — Submitted filename: Response for reviewers comments.docx [file pone.0328105.s019.docx]

**Response to editor and reviewers on “Meteorological drought under historical and future climate scenarios in North Gojjam sub-basin, Abay River basin of Ethiopia”**

**Manuscript Number**: PONE-D-24-44303R1

**Dear Editor: PLOS ONE**

Thank you for giving us the opportunity to submit a revised draft of our manuscript titled **Meteorological drought under historical and future climate scenarios in North Gojjam sub-basin, Abay River basin of Ethiopia** to *PLOS ONE.*

We appreciate the efforts that you and the reviewers have made to provide valuable feedback and suggestions to improve our manuscript. We are grateful to the reviewers for their insightful comments on the paper. We are delighted to have been able to respond to the suggestions provided by the reviewers. Below are point-by-point responses to the reviewers' comments. The reviewers' comments are in Italic font, and our responses are in **turquoise**.

**N.B:** In the revised manuscript, only the major corrections are highlighted in turquoise.

- The text highlighted in turquoise reflects changes made in response to the comments and suggestions of the reviewer

**Editor comments:**

*Since their response did not convience one of the reviewers, we invited another expert in the field to evalute the work. The authors need to provide point-by-point reponse to the review comments. Moreover, if any reviewer suggested citing a paper, you need to check if citing the suggested reference is improving your work or not. You can add the reference only if it improves your work. Otherwise, please provide a response to the raised comment.*

Response:

Dear editor,

Thank you for your comments. We have addressed all points raised by the academic editor and reviewers. A detailed point-by-point response is provided in the attached file. As requested, we have submitted both a clean and a tracked-changes version of the manuscript. Suggested references have been incorporated where relevant to the manuscript.

**Academic editor comments:** *The manuscript still needs to be signifincally improved as one of the reviewers still suggested rejection. When submitting your revised manuscript, provide a point-by-point response to all review comments so that a suitable decision is made for your article.*

**Response:** Dear Academic Editor, Thank you for the feedback. We have substantially revised the manuscript and addressed all reviewer comments in detail. A point-by-point response is provided to facilitate a thorough re-evaluation.

**Reviewer comments:**

***# 1****. In the Materials and Methods section, explain more about the Mann-Kendall method of routing and provide the relevant relationships. You can use the following resources for this.*

<https://doi.org/10.1016/B978-0-323-91910-4.00032-7>

<https://doi.org/10.3390/w16213077>

Response 1: Thank you for your valuable comments. We have now expanded the Materials and Methods section to provide a more detailed explanation of the Mann-Kendall method. In line with your suggestion, we have also included and cited the recommended source. Please refer to the revised section in the updated manuscript for the incorporation of your comments.

*#2. There are two tables with the number 3*

Response 2: Thank you for your constructive comment. We have corrected the issue of duplicate table numbering, and the revised manuscript now reflects the appropriate table numbers.

*#3. The format of Table 1 and 3 is not consistent with other tables.*

*Response 3: Thank you for your valuable comment. We have carefully reviewed and revised the formatting of Tables 1 and 3 to ensure consistency with the other tables in the revised manuscript*

\# 4. *To make things better, you can also calculate the uncertainty of temperature and precipitation data obtained from climate change scenarios, as in the reference below.*
[*https://doi.org/10.3390/hydrology11010002*](https://doi.org/10.3390/hydrology11010002)

Response: Thank you very much for your valuable suggestion regarding the calculation of uncertainty in temperature and precipitation data derived from climate change scenarios. We fully agree that uncertainty analysis is a crucial aspect of climate modeling. However, in the present study, we addressed model reliability and robustness using a comprehensive set of statistical validation metrics, including the correlation coefficient (R), Percent Bias (PBIAS), and Root Mean Square Error (RMSE). These methods were carefully selected to evaluate the model's performance across different scenarios.

\#*5. The abstract is too long, please summarize it.*

Response: Thank you for your comment. We have revised and shortened the abstract to provide a more concise summary of the manuscript in the revised version.

#6. In the section on calculating the SPEI index, show how to obtain this index in the form of relationships so that the reader can understand this topic at a glance
<https://dor.isc.ac/dor/20.1001.1.17352347.1398.15.4.24.7>

Response: Thank you for your constructive comment and valuable suggestion. In response, we have revised the manuscript to include a clear and concise explanation of the SPEI calculation process. The step-by-step formulation is now presented using mathematical expressions to help readers easily understand how the index is derived. Please refer to the updated manuscript for these revisions.

7. It would be better to also show past temperature and precipitation data and drought index in graphical form so that the reader can get a better understanding of the data trends.

Response: Thank you for your valuable suggestion. We have included temperature and precipitation trends in graphical form as supplementary figures. Due to the large number of figures already present in the manuscript, we have placed these additional graphs in the supplementary materials (S1 Fig. 1–S15 Fig.), which are included in the revised manuscript.

8. Lines 163 to 169 require references. You can use the following references.
<https://doi.org/10.1002/clen.201100652>
<https://doi.org/10.3390/hydrology9100176>

Response: Thank you for your valuable suggestion. We have revised the manuscript and incorporated the recommended reference to support Lines 120–126. The updated citations are now included on page 5, starting at line 126.

Thank you once again for your valuable feedback and for helping to improve our manuscript.

In addition to addressing the comments above, we have corrected all spelling and grammatical errors. Furthermore, as suggested by the editor, we have included the manuscript highlights.

We look forward to hearing from you in due course regarding our submission and are happy to respond to any further questions or comments you may have.

Thank you for your valuable and constructive comments and suggestions!
