# Supplementary material for: Meteorological drought under historical and future climate scenarios in North Gojjam sub-basin, Abay River basin of Ethiopia
Source: PLoS One. 2025 Jul 17;20(7):e0328105. doi: 10.1371/journal.pone.0328105 (PMC12270152; doi:10.1371/journal.pone.0328105)
Supplement: Attachment — Submitted filename: Minor Suggestions.docx [file pone.0328105.s020.docx]

**Dear Editor,**

I have reviewed the revised version of the manuscript and commend the authors for their comprehensive and thoughtful revisions. The authors have addressed all major concerns raised in previous review rounds with clarity and rigor. Methodological explanations, including the Mann-Kendall trend test, SPEI computation, bias correction procedures, and climate model selection, have been significantly improved. The manuscript now presents a clearer and more replicable study design, supported by appropriate references and well-structured analyses.

However, before final acceptance, I recommend that the authors carefully address the following **minor technical and editorial points:**

**Minor Revision:**

1- **Line 2**: “...climate scenarios in North Gojjam sub-basin, Abay River basin of Ethiopia”
   Use consistent spelling “Abay River Basin” (as used in the title). Standardize throughout the text.
2- Add a space between the number and unit: 24.7°C → 24.7 °C.
3- **Line 218:** Mention the specific packages used.
4- **Line 355:** “...where nnn covers the entire period.” Likely typo error.
5- Tables are not formatting well such as Table 8, 9 and 10, etc.
6- **Line 566**: “Run theory” is mentioned briefly. Consider a 1–2 sentence clarification or citation since not all readers may be familiar with it.
7- **Lines 358–365:** You state autocorrelation correction is applied but don't mention the lag selection or whether pre-whitening was used.
