# Supplementary material for: Meteorological drought under historical and future climate scenarios in North Gojjam sub-basin, Abay River basin of Ethiopia
Source: PLoS One. 2025 Jul 17;20(7):e0328105. doi: 10.1371/journal.pone.0328105 (PMC12270152; doi:10.1371/journal.pone.0328105)
Supplement: Attachment — Submitted filename: Response to Editor and Reviewers.docx [file pone.0328105.s021.docx]

**Response to Editor and Reviewers**

Manuscript ID: PONE-D-24-44303R3

Title: Meteorological drought under historical and future climate scenarios in North Gojjam sub-basin, Abay River basin of Ethiopia

**Dear Editor and Reviewers**

Thank you for the opportunity to revise and resubmit our manuscript to PLOS ONE. We would like to thank for the constructive feedback provided by the reviewers and the editorial team, which has helped us improve the clarity, rigor, and impact of our study. In this revised submission, we have addressed all editorial requirements and responded to each reviewer’s comments in detail*.*  We appreciate the efforts that you and the reviewers have made to provide valuable feedback and suggestions to improve our manuscript. We are grateful to the reviewers for their insightful comments on the paper. We are delighted to have been able to respond to the suggestions provided by the reviewers. Below are point-by-point responses to the reviewers' comments. The reviewers' comments are in Italic font. The sections highlighted in **turquoise** represent our responses to both the editor's and reviewers' feedback and indicate the corresponding revisions made in the main manuscript. All suggested changes have been incorporated into the revised manuscript, and we have uploaded all additional files as requested.

**Response to Editorial Requirements:**

We've checked your submission and before we can proceed, we need you to address the following issues:

We note that Figure 1 in your submission contain [map/satellite] images which may be copyrighted. All PLOS content is published under the Creative Commons Attribution License (CC BY 4.0), which means that the manuscript, images, and Supporting Information files will be freely available online, and any third party is permitted to access, download, copy, distribute, and use these materials in any way, even commercially, with proper attribution. For these reasons, we cannot publish previously copyrighted maps or satellite images created using proprietary data, such as Google software (Google Maps, Street View, and Earth). For more information, see our copyright guidelines: <http://journals.plos.org/plosone/s/licenses-and-copyright>.

We require you to either (1) present written permission from the copyright holder to publish these figures specifically under the CC BY 4.0 license, or (2) remove the figures from your submission:

a. You may seek permission from the original copyright holder of Figure 1 to publish the content specifically under the CC BY 4.0 license.

We recommend that you contact the original copyright holder with the Content Permission Form (<http://journals.plos.org/plosone/s/file?id=7c09/content-permission-form.pdf>) and the following text:
“I request permission for the open-access journal PLOS ONE to publish XXX under the Creative Commons Attribution License (CCAL) CC BY 4.0 (<http://creativecommons.org/licenses/by/4.0/>). Please be aware that this license allows unrestricted use and distribution, even commercially, by third parties. Please reply and provide explicit written permission to publish XXX under a CC BY license and complete the attached form.”

Please upload the completed Content Permission Form or other proof of granted permissions as an ""Other"" file with your submission. In the figure caption of the copyrighted figure, please include the following text: “Reprinted from [ref] under a CC BY license, with permission from [name of publisher], original copyright [original copyright year].”

b. If you are unable to obtain permission from the original copyright holder to publish these figures under the CC BY 4.0 license or if the copyright holder’s requirements are incompatible with the CC BY 4.0 license, please either i) remove the figure or ii) supply a replacement figure that complies with the CC BY 4.0 license. Please check copyright information on all replacement figures and update the figure caption with source information. If applicable, please specify in the figure caption text when a figure is similar but not identical to the original image and is therefore for illustrative purposes only.

The following resources for replacing copyrighted map figures may be helpful:
USGS National Map Viewer (public domain): <http://viewer.nationalmap.gov/viewer/>.

The Gateway to Astronaut Photography of Earth (public domain): <http://eol.jsc.nasa.gov/sseop/clickmap/>
Maps at the CIA (public domain): <https://www.cia.gov/library/publications/the-world-factbook/index.html> and <https://www.cia.gov/library/publications/cia-maps-publications/index.html>
NASA Earth Observatory (public domain): <http://earthobservatory.nasa.gov/>
Landsat: <http://landsat.visibleearth.nasa.gov/>

USGS EROS (Earth Resources Observatory and Science (EROS) Center) (public domain): [http://eros.usgs.gov/#](http://eros.usgs.gov/)
Natural Earth (public domain): <http://www.naturalearthdata.com/>

Response: *Thank you for your detailed and thoughtful feedback on our manuscript. We sincerely appreciate your careful review and your dedication to upholding high standards and open access principles.*

*In response to your concern regarding Figure 1, we would like to clarify that this figure is an original map created by the authors. The underlying shapefile data used to develop the map were freely downloaded from* [*OpenDataSoft*](https://public.opendatasoft.com)*, a public open data platform that provides spatial datasets for unrestricted use. As GIS and remote sensing experts, we routinely generate our own maps and figures using openly licensed, non-proprietary datasets, and we adhere to accepted cartographic and licensing standards.*

*To ensure full compliance with the Creative Commons Attribution (CC BY 4.0) license, we have updated the figure caption to clearly indicate the data source and confirm that the map does not incorporate any proprietary or copyrighted content (e.g., Google Maps or Google Earth). The revised caption now reads:*

***Fig 1.*** *Map of the study area developed by the authors. Source: Original shapefile data freely downloaded from* [*https://public.opendatasoft.com*](https://public.opendatasoft.com)*.*

\# Comment: Journal Requirements:

Please review your reference list to ensure that it is complete and correct. If you have cited papers that have been retracted, please include the rationale for doing so in the manuscript text, or remove these references and replace them with relevant current references. Any changes to the reference list should be mentioned in the rebuttal letter that accompanies your revised manuscript. If you need to cite a retracted article, indicate the article’s retracted status in the References list and also include a citation and full reference for the retraction notice.

Response: Thank you for your suggestions. As per the journal’s requirements, we have thoroughly reviewed all the references. The reference list is complete and accurate, and we confirm that no retracted papers have been cited.

**Reviewer comments:**

Reviewer 5:

***# 1****. Line 2: “...climate scenarios in North Gojjam sub-basin, Abay River basin of Ethiopia. Use consistent spelling “Abay River Basin” (as used in the title). Standardize throughout the text.*

Response 1: Thank you for your valuable comments. We have made the necessary corrections and ensured consistent spelling throughout the text (please refer the updated manuscript).

*#2. Add a space between the number and unit: 24.7°C → 24.7 °C.*

Response 2: Thank you for your constructive comment. We have addressed the issue and made the necessary corrections in the revised manuscript.

*#3. Line 218: Mention the specific packages used.*

*Response 3: We appreciate your insightful comment and have now included the specific packages used in the revised manuscript.*

\# 4.  *Line 355: “...where nnn covers the entire period.” Likely typo error.*

Response: Thank you for your valuable comment. We have corrected the typographic error.

\#*5. Tables are not formatting well such as Table 8, 9 and 10, etc.*

Response: Thank you for your comment. We have reviewed and corrected the formatting issues in the tables, including Tables 8, 9, and 10, to ensure consistency and clarity.

\#*6. Line 566: “Run theory” is mentioned briefly. Consider a 1–2 sentence clarification or citation since not all readers may be familiar with it.*

Response: Thank you for your constructive comment and valuable suggestion. We have now incorporated a clearer explanation of run theory to enhance understanding for all readers. Please refer to the updated manuscript.

7. *Lines 358–365: You state autocorrelation correction is applied but don't mention the lag selection or whether pre-whitening was used.*

Response: Thank you for pointing this out. In the revised manuscript, we have clarified that we applied a lag-1 autocorrelation correction, which is commonly used in drought and climate trend analyses.

We believe these revisions address all concerns and enhance the manuscript’s quality. Thank you again for your guidance. We look forward to your feedback!

Sincerely,

Dr. Tatek Belay

Corresponding Author

Debre Tabor University
